# Whole-Exome Sequencing (WES) Reveals Novel Sex-Specific Gene Variants in Non-Alcoholic Steatohepatitis (MASH)

**DOI:** 10.3390/genes15030357

**Published:** 2024-03-13

**Authors:** Jing Wei, Boyang Jason Wu, Sayed S. Daoud

**Affiliations:** Department of Pharmaceutical Sciences, College of Pharmacy and Pharmaceutical Sciences, Washington State University Health Sciences, Spokane, WA 99202, USA; jing.wei@wsu.edu (J.W.); boyang.wu@wsu.edu (B.J.W.)

**Keywords:** MASLD, MASH, sexual dimorphism, Wnt/β-catenin, *ALPK2* polymorphisms

## Abstract

Non-alcoholic steatohepatitis (NASH, also known as MASH) is a severe form of non-alcoholic fatty liver disease (NAFLD, also known as MASLD). Emerging data indicate that the progression of the disease to MASH is higher in postmenopausal women and that genetic susceptibility increases the risk of MASH-related cirrhosis. This study aimed to investigate the association between genetic polymorphisms in MASH and sexual dimorphism. We applied whole-exome sequencing (WES) to identify gene variants in 8 age-adjusted matched pairs of livers from both male and female patients. Sequencing alignment, variant calling, and annotation were performed using standard methods. Polymerase chain reaction (PCR) coupled with Sanger sequencing and immunoblot analysis were used to validate specific gene variants. cBioPortal and Gene Set Enrichment Analysis (GSEA) were used for actionable target analysis. We identified 148,881 gene variants, representing 57,121 and 50,150 variants in the female and male cohorts, respectively, of which 251 were highly significant and MASH sex-specific (*p* < 0.0286). Polymorphisms in *CAPN14*, *SLC37A3*, *BAZ1A*, *SRP54*, *MYH11*, *ABCC1*, and *RNFT1* were highly expressed in male liver samples. In female samples, Polymorphisms in *RGSL1*, *SLC17A2*, *HFE*, *NLRC5*, *ACTN4*, *SBF1*, and *ALPK2* were identified. A heterozygous variant 1151G>T located on 18q21.32 for *ALPK2* (rs3809983) was validated by Sanger sequencing and expressed only in female samples. Immunoblot analysis confirmed that the protein level of β-catenin in female samples was 2-fold higher than normal, whereas ALPK2 expression was 0.5-fold lower than normal. No changes in the protein levels of either ALPK2 or β-catenin were observed in male samples. Our study suggests that the perturbation of canonical Wnt/β-catenin signaling observed in postmenopausal women with MASH could be the result of polymorphisms in *ALPK2*.

## 1. Introduction

Non-alcoholic fatty liver disease (NAFLD) (also known as metabolic dysfunction-associated fatty liver disease, MAFLD) is the leading cause of chronic liver disease, affecting 25% of the US population [1,2]. It is commonly associated with obesity, diabetes, and metabolic syndrome but can also affect non-obese individuals. The disease spectrum ranges from bland steatosis with or without inflammation (non-alcoholic fatty liver, NAFL) to steatosis with inflammation and hepatocellular injury (non-alcoholic steatohepatitis, NASH) (also known as metabolic dysfunction-associated steatotic liver disease, MASH), fibrosis, cirrhosis, and hepatocellular carcinoma [3]. Owing to the lack of reliable noninvasive predictive biomarkers, the diagnosis of MASH is mainly limited to the histopathological evaluation of liver samples defined by liver-biopsy-proven hepatocellular steatosis, lobular inflammation, and evidence of hepatocyte injury such as ballooning degeneration [4]. A large body of evidence strongly supports the idea that MASLD susceptibility and progression to MASH are sex specific. Several studies conducted in single centers or in specific populations have suggested that women have a 19% lower risk of MASLD than men in the general population. However, once MASLD has become established, women have a 37% higher risk of advanced fibrosis than men [5]. Among individuals with established MASLD who are older than 50 years, women have a 17% greater risk for MASH and a 56% greater risk for advanced fibrosis than men [5,6]. Although it has been established that the prevalence of risk factors such as age, obesity, type 2 diabetes mellitus (T2DM), atherogenic dyslipidemia, and clinical outcomes of MASLD differs between sexes, the molecular mechanisms by which sex modulates the pathogenesis and clinical outcomes of MASLD progression are poorly defined. Therefore, to understand the potential mechanisms underlying this sexual dimorphism in MASLD prevalence, we recently used a multiomics approach with archived liver samples from both sexes to study the biological basis of the observed sexual dimorphism. Our study suggests (for the first time) that the activation of canonical Wnt signaling could be one of the main pathways associated with sexual dimorphism in MASLD and MASH [7].

Two different Wnt signaling pathways, canonical and non-canonical, have their own influence on MASLD and MASH. The non-canonical pathway is involved in the accumulation of fat, inflammation, and lipids, which promote MASH formation. The canonical pathway involving β-catenin functions as an anti-inflammatory, anti-lipid accretion, and adipocyte differentiation pathway [8]. Hence, the inhibition or downregulation of the classical Wnt/β-catenin pathway contributes to the onset and progression of MASLD. For example, MASLD is inhibited by the upregulation of peroxisome proliferator activated receptor γ (PPAR-γ), a downstream target of the Wnt/β-catenin signaling that promotes preadipocyte differentiation, adipogenesis, the absorption of free fatty acids (FFA), and the suppression of inflammation [9]. Polymorphisms in low-density lipoprotein receptor-related protein-6 (LRP6) are a major cause of MASLD [10]. Although it is well documented that MASLD progression is attributed to dynamic interactions between genetic and environmental factors [11], there is still limited information on how canonical Wnt/β-catenin signaling is involved in MASLD/MASH disease progression. Therefore, we hypothesized that gene variants in the Wnt/β-catenin signaling pathway could be associated with the observed sexual dimorphism in MASH, as suggested by our recent study [7].

To test this hypothesis, we used whole-exome sequencing (WES) to identify potential gene variants implicated in MASH using 16 archived frozen liver samples from paired males and females. Here, we report the identification of α protein kinase 2 (*ALPK2*) gene variants (rs3809981and rs3809983) as female-specific single-nucleotide polymorphisms (SNPs) in the postmenopausal livers of women with MASH.

## 2. Methods

### 2.1. Ethics Statement

The Institutional Review Board (IRB) of Washington State University (WSU) approved the protocol of the current study. Sixteen paired matched snap-frozen tissue samples were obtained from the IRB-approved University of Minnesota Liver Tissue Cell Distribution System (LTCDS). All specimens with anonymized identifiers were histopathologically confirmed by a pathologist (Table S1; Supplemental Digital Content).

### 2.2. DNA Extraction and Whole-Exome Sequencing (WES-Seq)

Genomic DNA was extracted from 16 frozen liver tissue samples (4 matched pairs of both sexes) using a Wizard Genomic DNA purification kit (A1120, Promega, Madison, WI, USA) following the manufacturer’s instructions. The DNA concentration was measured using a NanoDrop spectrophotometer (Thermo Fisher Scientific, Waltham, MA, USA). The extracted DNA (50 ng/uL/sample) was shipped to LC Sciences (Houston, TX, USA) for exome sequencing (100× coverage). Two hundred nanograms of genomic DNA (200 ng) from each subject’s MASH-normal paired samples, which were fragmented by sonication, were subjected to library preparation using the Agilent SureSelect Human All Exon V6 kit (Agilent Technologies, Santa Clara, CA, USA) following the vendor’s recommended protocol. DNA libraries were hybridized and captured using SureSelect. Following hybridization, the captured libraries were purified according to the manufacturer’s instructions and amplified by polymerase chain reaction (PCR). Normalized libraries were pooled, and DNA was subjected to paired-end sequencing using the Illumina HiSeq X Ten platform with a 150-bp paired-end sequencing mode.

### 2.3. WES Data Processing

Raw sequence reads were trimmed to remove low-quality sequences and then aligned to the human reference genome (hg19) using the Burrows–Wheeler alignment tool [12]. Single-nucleotide polymorphisms and small insertions/deletions were identified in individual samples using the Genome Analysis Toolkit (GATK Mutect2 4.0.4.0) with the default setting [13]. ANNOVAR was then used to annotate the VCF files using the gene region and several filters from other databases [14]. Finally, we used the Database for Annotation, Visualization, and Integrated Discovery (DAVID) Bioinformatics Resource 6.7 (https://David-d.ncifcrf.gov, accessed on 22 July 2023) and Gene Set Enrichment Analysis (GESA) [15] to identify significantly altered biological processes and pathways in 16 liver tissue samples.

### 2.4. PCR and Sanger Sequencing

To validate the *ALPK2* polymorphisms, we used PCR and Sanger sequencing from Azenta Life Sciences (Burlington, MA, USA). Specific PCR primers for *ALPK2,* F: TGCTGTCTATCAAATCTCGGCT and R: GAGCACTCAACCTCAACGGA were used. Primers were designed using Primer3 (http://bioinfo.ut.ee/primer3-0.4.0/, accessed on 22 July 2023). The products were directly sequenced using the ABI PRISM BigDye Kit on an ABI 3130 DNA sequencer (Applied Biosystems, Foster City, CA, USA). Sequencing results were analyzed using A Plasmid Editor [16].

### 2.5. Western Blot Analysis

Frozen liver tissue samples (n = 12) were homogenized in ice-cold lysis buffer containing a protease/phosphatase inhibitor cocktail and centrifuged at 12,000× *g* at 4 °C for 15 min. Protein samples were separated by sodium dodecyl sulfate–polyacrylamide gel electrophoresis (SDS-PAGE) and transferred onto polyvinylidene difluoride (PVDF) membranes. After blocking in 5% non-fat milk at 37 °C for 1 h, the membranes were incubated overnight at 4 °C with primary antibodies against ALPK2 (ab111909, Abcam, Cambridge, UK), β-catenin (8480S, Cell Signaling Technology, Danvers, MA, USA), or GAPDH (sc-47724, Santa Cruz Biotechnology, Dallas, TX, USA). Following incubation with the secondary antibody, immunoreactive proteins were visualized using the ChemiDoc Touch Imaging System (Bio-Rad). Protein bands were quantified using the ImageJ 1.53k.

### 2.6. Statistical Analysis of Western Blot

The data were expressed as the mean ± SEM (n = 3/phenotype/sex) and Student’s *t*-test was used to analyze statistical significance. Statistical analyses were performed, and graphs were generated using GraphPad Prism 6 (GraphPad Software Inc., San Diego, CA, USA). ** *p* < 0.01 was considered statistically significant.

## 3. Results

### 3.1. Clinical Characteristics of the Study Population

Sixteen snap-frozen liver tissue samples (normal and MASH) from white non-Hispanic populations of both sexes were used in this study. The median age (range) of patients was 54 to 59 years old. In general, the clinicopathological characteristics of patients with MASH (steatosis, steatohepatitis, ballooning, and portal inflammation) were higher in women than in men. Detailed clinicopathological information is summarized in Supplemental Table S1.

### 3.2. WES, Data Filtering and Mutation Landscape of Liver Tissue Samples

**Figure 1:**
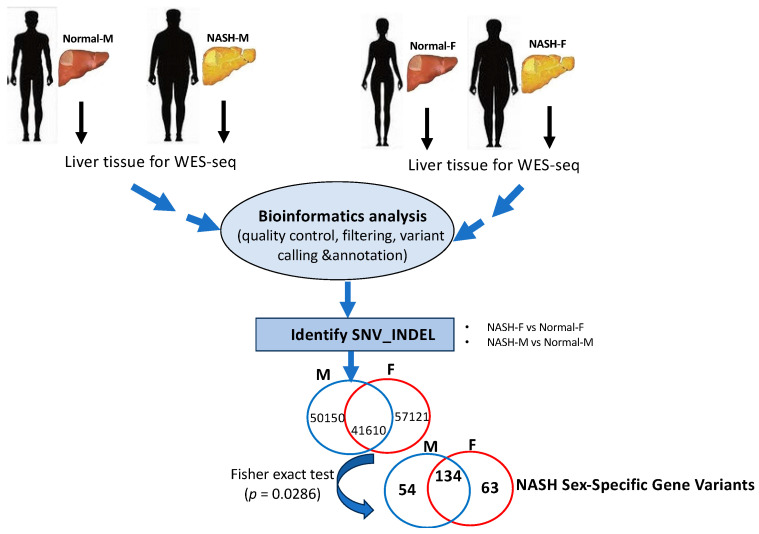
Illustration of WES workflow from frozen liver tissue samples of male and female patients to MASH sex-specific gene variants. Pipeline of bioinformatics analysis adapted in the WES results of gene variants.

**Figure 2:**
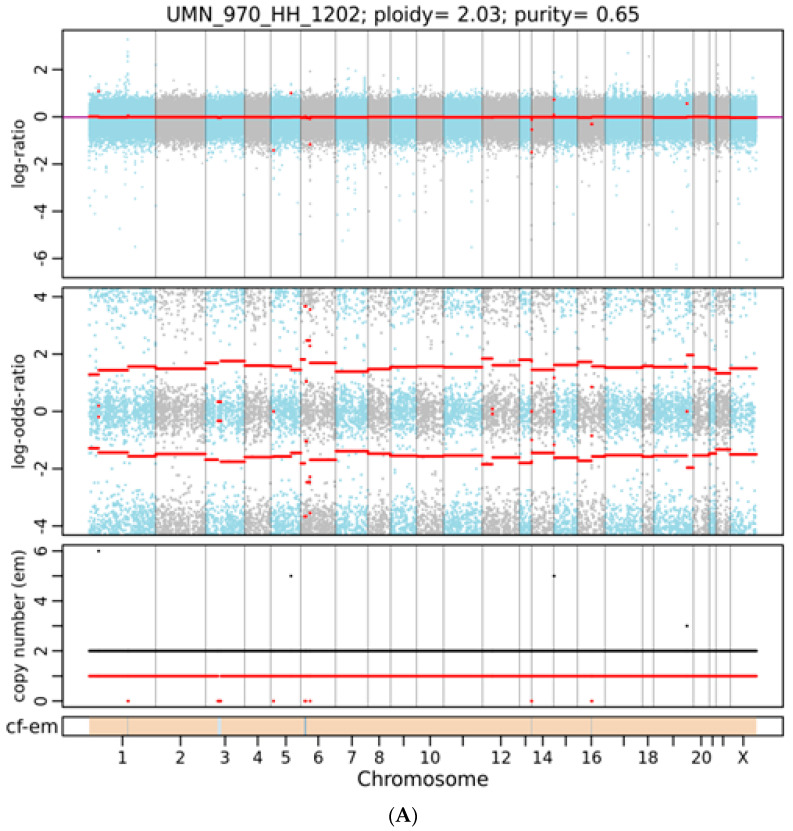

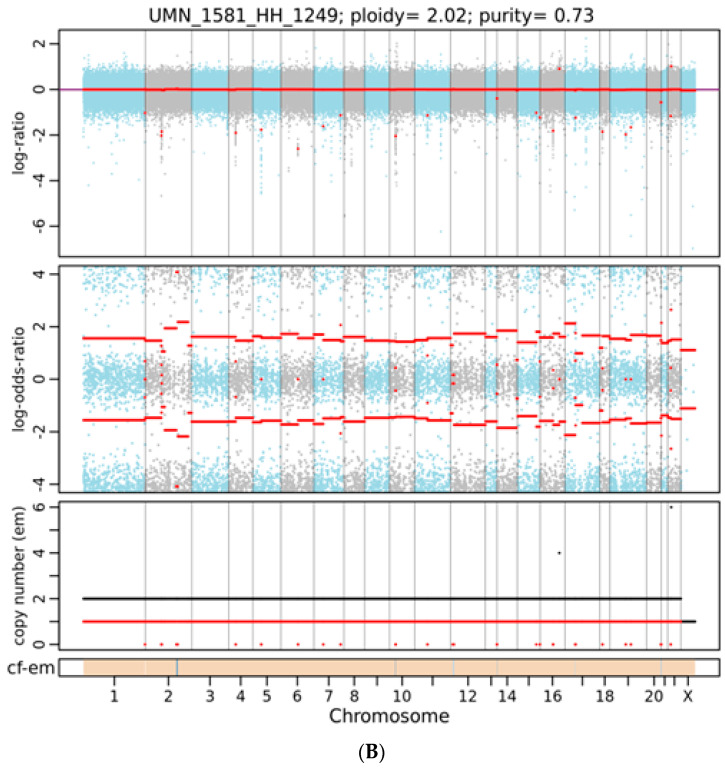
A representative integrated visualization of FACETS analysis of WES data for (**A**) female and (**B**) male total copy number variants (CNVs). The top panel displays total copy number log-ratio (logR), and the second panel displays allele-specific log-odds-ratio data (logOR) with chromosomes alternating in blue and gray. The third panel plots the corresponding integer (total, minor) copy number calls. The overall ploidy and purity for female patients in this case are 2.03 and 0.65, respectively, and 2.05 and 0.63 for male patients. The estimated cellular fraction (cf) profile is plotted at the bottom, revealing the aggregate of variants at each chromosome.

As shown in Figure 1, using the WES approach we identified 148,881 gene variants in 16 liver tissue samples, representing 57,121 and 50,150 gene variants in female and male cohorts, respectively. For SNVs, 35,000 (27%) were exonic and 79,259 (59%) were intronic (Table 1). For InDels, 13,925 were identified and 10,837 (78%) were intronic, as shown in Table 1. Our analysis detected no differences in SNPs, InDel distribution, or mutation type between sexes (Supplemental Figures S1 and S2). By contrast, FACETS analysis [17] revealed that copy number variants (CNVs) in female cohorts differed from those in male cohorts. As shown in Figure 2A, many gene variants (female cases), such as *SLC17A2* (Table 2), were clustered around chromosome 6 (as represented by allele-specific log-odd-ratio data), whereas in male cases (Figure 2B), many gene variants such as *CAPN14* (Table 3) were clustered around chromosome 11. Collectively, these observations suggest that copy-number alterations (CNAs) of these genes are different in the two cohorts and could play an important role in the sexual dimorphism of MASH.

### 3.3. WES Identifies ALPK2 Variant in Female Cases

To further analyze our gene variant data, statistical significance was first determined via a hypothetical Fisher’s exact test (Figure 1); with four male samples vs. four females, a polymorphism was considered significant if it existed in all female samples but none of the male samples (or if a polymorphism existed in all male samples but none of the female samples). The corresponding *p*-value for this assumption was 0.0286. We merged and filtered the vcf files of individual samples and searched for polymorphisms that met the above criteria. Polymorphisms that passed the criteria were then annotated with the Var2GO tool [18], using GRCh37 as a reference, given that the original analysis was performed using the hg19 genome. A total of 251 highly significant sex-specific MASH gene variants (*p* < 0.0286) were identified. A total of 63 MASH female-specific gene variants were identified, as shown in Table 2, whereas 54 gene variants were identified in males (Table 3). Among the 54 male variants, we found polymorphisms in *CPN14* (12 intronic variants), *SRP54* (four intronic and upstream gene variants), *ABCC1* (three synonymous and intronic gene variants), *RNFT1* (two upstream gene variants), *SLC37A3* (two intronic and upstream gene variants), obg-like ATPase 1 (*OLA1*) (two intronic and non-synonymous variants), *BAZ1A* (two intronic and downstream gene variants), and *MYH11* (two intronic and synonymous variants). 

Of the 63 female variants (Table 2), we identified *SCL17A* (six intronic and synonymous variants), *RGSL1* (three intronic variants), *ACTN4* (three synonymous and upstream gene variants), *NLRC5* (two synonymous and non-synonymous variants), *BIN1* (two intronic variants), *C7* (two intronic and downstream gene variants), *HIST1H4B* (synonymous and downstream gene variants), *SBF1* (two upstream gene variants), and *ALPK2* (two synonymous and non-synonymous variants). In this study, we validated α Protein Kinase 2 (ALPK2) as a novel genetic variant associated with MASH in a female cohort.

### 3.4. Validation of the ALPK2 Variant

To identify the biological pathways associated with *ALPK2*, we performed gene set enrichment analysis (GSEA) [15] using a TCGA liver cancer patient cohort from the cBioPortal database. As shown in Table 4, Wnt gene signatures, including canonical/β-catenin-mediated pathways, were negatively enriched in *ALPK2*-high (FDR q-val = 0.036 to 0.003) vs. *ALPK2*-low samples (FDR q-val = 0.105 to 0.544), which is consistent with a previous report showing ALPK2 as a negative regulator of canonical Wnt signaling [19]. These data also confirmed that ALPK2 is associated with β-catenin-mediated pathways in women with MASH, as we previously reported [7].

**Figure 3:**
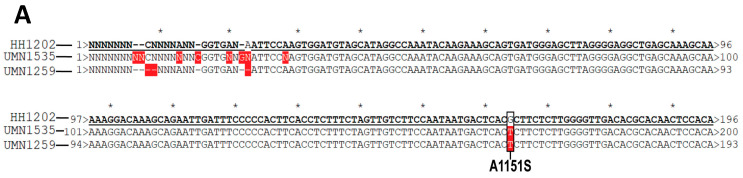

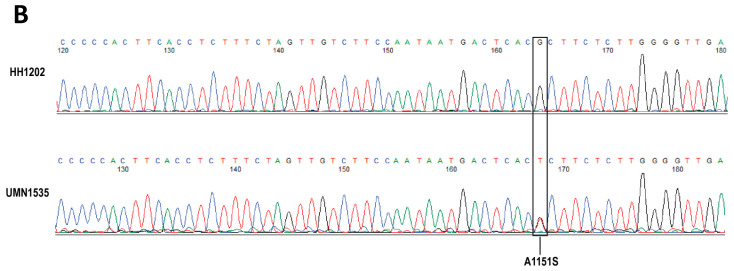
Representative of Sanger sequence alignment (**A**) and chromatograms (**B**) of ALPK2 in normal and MASH female livers. Sequencing alignment was performed using a plasmid editor. A normal representative liver sample with no SNPs (HH1202) was used as a reference for comparison against two MASH-related samples (UMN1535 and UMN1259). A clear SNP is highlighted with a black box. The SNP leads to a substitution mutation from a hydrophobic alanine (A) at the 1151 position to a polar serine (S). No SNPs were observed in MASH-related samples of male patients.

Next, we validated the *ALPK2* mutation by PCR testing coupled with Sanger sequencing. As shown in Figure 3, the normal, healthy sample HH1202 was used as a reference for comparison with the two female MASH samples (UMN1535 and UMN1259). A clear single nucleotide polymorphism (SNP) is highlighted with a black box in the MASH samples in Figure 3A,B. The identified SNP (p.Ala1551Ser) resulted in nsSNV (rs3809983), as shown in Table 2.

**Figure 4:**
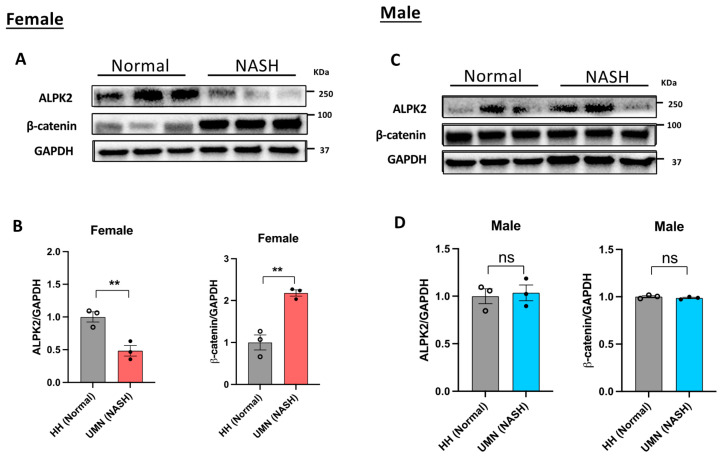
Immunoblot analysis of ALPK2 and β-catenin in female (**A**,**B**) and male (**C**,**D**) liver tissue samples. ALPK2 and β-catenin protein band intensity results were normalized to GAPDH and quantitatively analyzed with ImageJ 1.53k.. The ratio of target protein to GAPDH in individual normal groups was set as 1. Data represent the mean ± SEM. ** *p* < 0.01; ns, not significant; n = 3 samples/phenotype/sex.

Since *ALPK2* was shown to be involved in the canonical Wnt/β-catenin signaling pathway (Table 4), we measured the protein expression of both ALPK2 and β-catenin in both male and female liver tissue samples using immunoblot analysis. As shown in Figure 4A,B, the protein expression of β-catenin in female samples was 2-fold higher than that in normal samples, whereas ALPK2 expression was 0.5-fold lower than that in normal samples. No change in the expression of either ALPK2 or β-catenin was observed in male samples (Figure 4C,D).

## 4. Discussion

Genetics play a key role in MASLD pathogenesis [20,21]. Variations in genes such as patatin-like phospholipase domain-containing protein 3 (*PNPLA3*), transmembrane 6 superfamily member 2 (*TM6SF2*), membrane-bound O-acyltransferase domain-containing 7 (*MBOAT7*), glucokinase regulator (*GCKR*), and hydroxysteroid 17-β dehydrogenase-13 (*HSD17B13*) have emerged as reproducibly and robustly predisposing individuals to the development of MASH [22,23]. However, despite these discoveries, some unexplained variance remains, indicating that additional genetic associations with MASLD/MASH may be revealed using multi-omics analyses.

Although sex differences exist in the prevalence, risk factors, fibrosis, and clinical outcomes of MASLD/MASH, our understanding of the genetic basis of sexual dimorphism remains limited. Therefore, in this study, we performed WES analyses of paired-matched liver tissue samples from male and female MASH patients (Table S1) to elucidate sex-specific gene variants associated with this disease. As shown in Figure 1, we identified 63 gene variants that were specific to the female and 54 male-specific variants (Fisher’s exact test *p* < 0.0286). Interestingly, a significant number of these gene variants have been identified with respect to the sexual dimorphism of MASLD/MASH, whereas others have been previously reported to be involved in the pathogenesis of the disease. For example, in male-specific variants (Table 3), we identified *CAPN14* as encoding a calcium-regulated non-lysosomal thiol-protease (Calpain) as a top gene variant that is known to be involved in a variety of cellular processes including apoptosis, cell division, the modulation of integrin–cytoskeletal interactions, and synaptic plasticity [24]. Recently, calpains have been shown to be associated with hepatocyte death in MASH and the progression of hepatocellular carcinoma (HCC) [25,26]. Regarding chr2 (2p23.1), we found that *OLA1* encodes a member of the GTPase protein family. It interacts with breast-cancer-associated gene 1 (BRCA1) and BRCA1-associated RING domain protein (BRAD1) and is involved in centrosome regulation [27]. *OLA1* has been shown to be associated with hereditary breast and ovarian cancers as well as with a poor prognosis of HCC [28,29]. Polymorphisms were also found in other canonical cancer-related genes, including *SLC37A3*, *BAZ1A*, *SRP54*, *MYH1* and *ABCC1* [30,31,32,33], but were not directly involved in MASLD pathogenesis. As shown in Table 3, we also identified a SNP (synonymous variant) in *ORAI1* (ORAI calcium release-activated calcium modulator 1), which encodes a membrane calcium channel subunit activated by the calcium sensor STIM1 when calcium stores are depleted [34]. *ORAI* polymorphisms have been shown to be associated with non-canonical Wnt signaling, MASLD progression, and HCC [35,36]. 

For female-specific gene variants (Table 2), we identified six loci of *SLC17A2* on chr6 (6p22.2), encoding proteins belonging to sodium-dependent phosphate transporters. A recent study reported that *SLC17A2* variants were associated with MASLD in lean individuals [37]. In the present study, *SLC17A2* was specifically identified in female MASH patients. For the same chr6 (6p22.2), we also established that *HFE* encodes a transmembrane protein that regulates iron absorption by regulating the interaction of the transferrin receptor with transferrin associated with MASLD in lean individuals along with *SLC17A2* (37). For chr16 (16q13), we identified two loci *NLRC5* that encode members of the caspase recruitment domain of the NLR family. This gene plays a major role in the regulation of the NF-kappa B and interferon signaling pathways [38]. Polymorphisms in *NLRC5* are associated with obesity, type 2 diabetes mellitus (T2DM), and MASLD [39] and limit the NF-kB signaling pathway [40].

In the present study, we identified rs3809983 *ALPK2* as a novel gene variant associated with MASH in female liver samples. *ALPK2* mapped to 18q21.32 encodes a serine/threonine kinase protein that is involved in several processes, including epicardium morphogenesis and heart development, and is a negative regulator of Wnt signaling [19]. Recent studies by McIntosh et al. [41] showed that *ALPK2* rs3809973 (not *ALPK2* rs3809983, identified in this study) is associated with an increased risk of liver fibrosis in HIV/HCV co-infected women. This may be the initial indication linking the *ALPK2* variant to the pathological liver phenotype in women. Furthermore, Lawrence et al. [42] found that *ALPK2* is a novel polymorphic gene in human cancers in a large-scale genomic analysis of 4742 human neoplasms and their matched normal tissue samples. In mouse xenograft models, the knockdown of *ALPK2* inhibits the development and progression of ovarian cancer [43] and renal cancer cells [44], thus supporting its relevance not only in cancer initiation and development but also in the pathogenesis of liver disease.

To validate the *ALPK2* polymorphism, we used PCR coupled with Sanger sequencing and found that *ALPK2* rs3809983 was associated with MASH in the female patient samples (Figure 3). This association was further confirmed by immunoblot analysis (Figure 4), suggesting that the *ALPK2* polymorphism was linked to defective canonical Wnt signal transduction only in female samples. *ALPK2* polymorphisms cause inappropriate levels of β-catenin and thus a perturbation of the Wnt signaling pathway in female patients with MASH, as we previously reported [7]. These observations thus agree with the cBioPortal analysis (Table 4), suggesting a good correlation between *ALPK2* loss/decreased function and the loss of its negative regulatory activity in the canonical Wnt/β-catenin signaling pathway.

Despite the important findings of this study, it has some limitations. These limitations are primarily associated with the availability of paired matched MASLD/MASH liver samples from the male and female cohorts. Although the present study was limited by the relatively small number of available samples, the data presented here showed a clear and robust distinction between female and male patients with respect to gene variants associated with MASH livers compared with normal livers. We hypothesize that future efforts should be made to increase the sample size while improving the selection of extreme phenotypes to maximize the power of this strategy. Demographic variables such as ethnic background should be considered in future studies. Owing to sample availability, the individuals included in our study were mainly of Caucasian origin, which may limit the applicability of our findings to other ethnic populations. These limitations highlight the critical need to improve research in this area, especially in clinically relevant conditions associated with MASLD and MASH such as inter-hepatic cholangiocarcinoma and celiac disease [45,46]. Further studies are also needed to elucidate the cellular and molecular basis on how *ALPK2* variants may impact the sexual dimorphism of MASLD/MASH disease progression. 

In summary, this study provides evidence that MASLD-related sexual dimorphism is influenced by genetic variants. We used WES of the liver tissue samples to identify sex-specific gene polymorphisms associated with MASH. Our study further provides evidence that polymorphisms in *ALPK2* are associated with postmenopausal women compared to men and that the activation of the canonical Wnt signaling pathway previously reported [7] could be the result of *ALPK2* polymorphisms. Other (downstream) members of the Wnt signaling pathway could also be associated with MASH severity in postmenopausal women compared to men.

## Figures and Tables

**Table 1:** Statistics of somatic SNV and InDels in position.

Type	SNV	InDels
	Counts Percent	Counts Percent
Downstream	580 0.4	72 0.5
Exonic	35,000 27	733 5
Exonic; splicing	17 0	4 0
Intergenic	3941 3	371 3
Intronic	79,259 59	10,837 78
ncRNA_exonic	3011 2	252 2
ncRNA_intronic	4444 3	550 4
ncRNA_splicing	8 0	0 0
Splicing	111 0.1	47 0.3
Upstream	1228 1	115 1
Upstream; downstream	115 0.1	5 0
UTR3	4504 3	687 5
UTR5	2730 2	247 2
UTR5; UTR3	13 0	5 0
All	134,961 100	13,925 100

SNV = s ingle nucleotide variant; InDels = insertion/deletion mutations.

**Table 2:** Female common uniquely significant annotated variants.

#CHR OM	POS	Gene_ID	Gene_Name	CytoBand	Avsnp150	Category	REF	ALT	Gene_Full_Name	*p*-Value
chr1	17085589	ENSG00000186715	*MST1L*	1p36.13	rs3863807	upstream_gene_variant	AGC GCTG	A	macrophage stimulating 1-like	0.0286
chr1	26487940	ENSG00000197245	*FAM110D*	1p36.11	rs3748856	missense_variant	A	G	family with sequence similarity 110 member D	0.0286
chr1	26496455	ENSG00000142684	*ZNF593*	1p36.11	rs2232648	5_prime_UTR_premature _start_codon_gain_variant	C	T	zinc finger protein 593	0.0286
chr1	154941593	ENSG00000160691	*SHC1*	1q21.3	rs4845401	upstream_gene_variant	C	G	SHC (Src homology 2 domain containing) transforming protein 1	0.0286
chr1	182509292	ENSG00000121446	*RGSL1*	1q25.3	rs266531	intron_variant	A	G	regulator of G-protein signaling like 1	0.0286
chr1	182509617	ENSG00000121446	*RGSL1*	1q25.3	rs3911280	intron_variant	C	A	regulator of G-protein signaling like 1	0.0286
chr1	182517357	ENSG00000121446	*RGSL1*	1q25.3	rs6657620	intron_variant	G	C	regulator of G-protein signaling like 1	0.0286
chr1	232172374	ENSG00000162946	*DISC1*	1q42.2	rs17773715	intron_variant	G	A	TSNAX-DISC1 readthrough (NMD candidate)	0.0286
chr2	55176112	ENSG00000214595	*EML6*	2p16.1	rs13394146	intron_variant	C	T	echinoderm microtubule associated protein like 6	0.0286
chr2	84668155	ENSG00000163541	*SUCLG1*	2p11.2	rs115384987	downstream_gene_variant	T	C	succinate-CoA ligase, α subunit	0.0286
chr2	127808226	ENSG00000136717	*BIN1*	2q14.3	rs2071270	intron_variant	A	T	bridging integrator 1	0.0286
chr2	127821085	ENSG00000136717	*BIN1*	2q14.3	rs2071268	intron_variant	C	T	bridging integrator 1	0.0286
chr2	202526366	ENSG00000082126	*MPP4*	2q33.1	rs62193397	downstream_gene_variant	G	A	membrane protein, palmitoylated 4	0.0286
chr3	32933360	ENSG00000206557	*TRIM71*	3p22.3	rs372794141	3_prime_UTR_variant	C	T,CTT	tripartite motif containing 71, E3 ubiquitin protein ligase	0.0286
chr3	57431721	ENSG00000559559	*DNAH12*	3p14.3	rs372891308	missense_variant	AAAAT	A	dynein axonemal heavy chain 12	0.0286
chr4	110896050	ENSG00000138798	*EGF*	4q25	rs2067004	sequence_feature	A	C	epidermal growth factor	0.0286
chr5	40980086	ENSG00000112936	*C7*	5p13.1	rs1450664	splice_region_variant and intron_variant	T	C	complement component 7	0.0286
chr5	40981689	ENSG00000112936	*C7*	5p13.1	rs1061429	3_prime_UTR_variant	C	A	complement component 7	0.0286
chr6	25914801	ENSG00000112337	*SLC17A2*	6p22.2	rs62394272	missense_variant	G	A	solute carrier family 17 member 2	0.0286
chr6	25914901	ENSG00000112337	*SLC17A2*	6p22.2	rs2071298	splice_region_variant and intron_variant	G	A	solute carrier family 17 member 2	0.0286
chr6	25916979	ENSG00000112337	*SLC17A2*	6p22.2	rs1865760	synonymous_variant	C	T	solute carrier family 17 member 2	0.0286
chr6	25918688	ENSG00000112337	*SLC17A2*	6p22.2	rs1865760	intron_variant	G	A	solute carrier family 17 member 2	0.0286
chr6	25924158	ENSG00000112337	*SLC17A2*	6p22.2	rs1540273	intron_variant	T	C	solute carrier family 17 member 2	0.0286
chr6	25925823	ENSG00000112337	*SLC17A2*	6p22.2	rs7770139	intron_variant	A	G	solute carrier family 17 member 2	0.0286
chr6	26027135	ENSG00000124529	*HIST1H4B*	6p22.2	rs3752420	3_prime_UTR_variant	G	A	histone cluster 1, H4b	0.0286
chr6	26027433	ENSG00000124529	*HIST1H4B*	6p22.2	rs3752419	synonymous_variant	G	A	histone cluster 1, H4b	0.0286
chr6	26087856	ENSG00000010704	*HFE*	6p22.2	rs2858993	intron_variant	T	A	homeostatic iron regulator	0.0286
chr6	71011831	ENSG00000112280	*COL9A1*	6q13	rs2242589	intron_variant	C	T	collagen type IX α 1	0.0286
chr6	99819556	ENSG00000132423	*COQ3*	6q16.2	rs4574651	downstream_gene_variant	C	T	coenzyme Q3 methyltransferase	0.0286
chr6	152679729	ENSG00000131018	*SYNE1*	6q25.2	rs9478326	intron_variant	G	A	spectrin repeat containing nuclear envelope 1	0.0286
chr7	142498813	ENSG00000211772	*TRBC2*	7q34	rs1042955	synonymous_variant	G	A	T cell receptor β constant 2	0.0286
chr8	103301555	ENSG00000104517	*UBR5*	8q22.3	rs2168689	intron_variant	T	C	ubiquitin protein ligase E3 component n-recognin 5	0.0286
chr9	107593182	ENSG00000165029	*ABCA1*	9q31.1	rs4743763	intron_variant	A	T	ATP binding cassette subfamily A member 1	0.0286
chr10	47701275	ENSG00000198250	*ANTXRL*	10q11.22	rs10906952	synonymous SNV	G	A	anthrax toxin receptor-like	0.0286
chr10	126480381	ENSG00000203791	*METTL10*	10q26.13	rs965484	missense_variant	C	T	EEF1A lysine methyltransferase 2	0.0286
chr11	72309540	ENSG00000186642	*PDE2A*	11q13.4	rs4943939	upstream_gene_variant	C	T	phosphodiesterase 2A	0.0286
chr12	9750669	ENSG00000111796	*KLRB1*	12p13.31	rs1135816	nonsynonymous SNV	A	G	killer cell lectin like receptor B1	0.0286
chr12	53880122	ENSG00000139625	*MAP3K12*	12q13.13	rs3816806	upstream_gene_variant	T	C	mitogen-activated protein kinase 12	0.0286
chr12	53896984	ENSG00000139546	*TARBP2*	12q13.13	rs2280448	3_prime_UTR_variant	G	A	TAR (HIV-1) RNA binding protein 2	0.0286
chr12	56865338	ENSG00000135423	*GLS2*	12q13.3	rs2657879	nonsynonymous SNV	A	G	glutaminase 2	0.0286
chr12	56866334	ENSG00000135517	*MIP*	12q13.3	rs2657880	upstream_gene_variant	T	A	major intrinsic factor of lens fiber	0.0286
chr12	88448328	ENSG00000133641	*C12orf29*	12q21.32	rs17418744	downstream_gene_variant	T	A	centrosomal protein 290kDa	0.0286
chr12	119419632	ENSG00000139767	*SRRM4*	12q24.23	rs1568924	5_prime_UTR_variant	C	T	serine/arginine repetitive matrix 4	0.0286
chr14	65414976	ENSG00000139998	*RAB15*	14q23.3	rs11540871	3_prime_UTR_variant	C	T	RAB15, member RAS oncogene family	0.0286
chr14	71215822	ENSG00000006432	*MAP3K9*	14q24.2	rs79518608	downstream_gene_variant	T	C	mitogen-activated protein kinase 9	0.0286
chr14	105268104	ENSG00000179627	*ZBTB42*	14q32.33	rs10141867	synonymous_variant	G	A	zinc finger and BTB domain containing 42	0.0286
chr14	107211211	ENSG00000211976	*IGHV3-73*	14q32.33	rs2073668	synonymous_variant	G	A	immunoglobulin heavy variable 3-73	0.0286
chr16	57075379	ENSG00000140853	*NLRC5*	16q13	rs35622257	missense_variant	G	GT	NLR family, CARD domain containing 5	0.0286
chr16	57080528	ENSG00000140853	*NLRC5*	16q13	rs289723	nonsynonymous SNV	C	A	NLR family, CARD domain containing 5	0.0286
chr17	12832063	ENSG00000006740	*ARHGAP44*	17p12	rs1317990	intron_variant	G	T	Rho GTPase activating protein 44	0.0286
chr17	76867017	ENSG00000035862	*TIMP2*	17q25.3	rs2277698	synonymous_variant	C	T	TIMP metallopeptidase inhibitor 2	0.0286
chr18	56202768	ENSG00000198796	*ALPK2*	18q21.32	rs3809983	nonsynonymous SNV	C	A	α kinase 2	0.0286
chr18	56203120	ENSG00000198796	*ALPK2*	18q21.32	rs3809981	synonymous_variant	C	T	α kinase 2	0.0286
chr18	77724726	ENSG00000226742	*HSBP1L1*	18q23	rs8095764	5_prime_UTR_variant	A	C	heat shock factor binding protein 1-like 1	0.0286
chr19	17091368	ENSG00000160111	*CPAMD8*	19p13.11	rs8103646	synonymous_variant	T	G	C3- and PZP-like, α-2-macroglobulin domain containing 8	0.0286
chr19	39138608	ENSG00000130402	*ACTN4*	19q13.2	rs2303040	upstream_gene_variant	T	C	actinin α 4	0.0286
chr19	39196745	ENSG00000130402	*ACTN4*	19q13.2	rs3745859	synonymous SNV	C	T	actinin α 4	0.0286
chr19	39215333	ENSG00000130402	*ACTN4*	19q13.2	rs3786851	upstream_gene_variant	C	T	actinin α 4	0.0286
chr19	55644442	ENSG00000105048	*TNNT1*	19q13.42	rs891186	downstream_gene_variant	G	A	troponin T1, slow skeletal type	0.0286
chr20	1617069	ENSG00000089012	*SIRPG*	20p13	rs2277761	synonymous_variant	A	G	signal regulatory protein γ	0.0286
chr22	29834766	ENSG00000128250	*RFPL1*	22q12.2	rs465736	5_prime_UTR_variant	A	G	RFPL1 antisense RNA 1	0.0286
chr22	50906518	ENSG00000100241	*SBF1*	22q13.33	rs1983679	upstream_gene_variant	G	A	SET binding factor 1	0.0286
chr22	50906917	ENSG00000100241	*SBF1*	22q13.33	rs9616852	upstream_gene_variant	C	A	SET binding factor 1	0.0286
chrX	149937404	ENSG00000102181	*CD99L2*	Xq28	rs41311690	3_prime_UTR_variant	T	C	CD99 molecule-like 2	0.0286

**Table 3:** Male common uniquely significant annotated variants.

#CHR OM	POS	Gene_ID	Gene_Name	CytoBand	Avsnp150	Category	REF	ALT	Gene_Full_Name	*p*-Value
chr1	114515717	ENSG00000163349	*HIPK1*	1p13.2	rs2358996	synonymous_variant	G	A	homeodomain interacting protein kinase 1	0.0286
chr1	234573357	ENSG00000059588	*TARBP1*	1q42.2	rs2273875	intron_variant	G	C	TAR (HIV-1) RNA binding protein 1	0.0286
chr1	237817784	ENSG00000198626	*RYR2*	1q43	rs669375	intron_variant	A	G	ryanodine receptor 2	0.0286
chr2	31397696	ENSG00000214711	*CAPN14*	2p23.1	rs10180369	intron_variant	G	C	calpain 14	0.0286
chr2	31397727	ENSG00000214711	*CAPN14*	2p23.1	rs10180369	intron_variant	T	C	calpain 14	0.0286
chr2	31399659	ENSG00000214711	*CAPN14*	2p23.1	rs6720151	intron_variant	T	C	calpain 14	0.0286
chr2	31399751	ENSG00000214711	*CAPN14*	2p23.1	rs6720254	intron_variant	T	G	calpain 14	0.0286
chr2	31399988	ENSG00000214711	*CAPN14*	2p23.1	rs4592896	non-synonymous SNV	C	T	calpain 14	0.0286
chr2	31400039	ENSG00000214711	*CAPN14*	2p23.1	rs4516476	intron_variant	A	G	calpain 14	0.0286
chr2	31400502	ENSG00000214711	*CAPN14*	2p23.1	rs13421721	intron_variant	A	C	calpain 14	0.0286
chr2	31400510	ENSG00000214711	*CAPN14*	2p23.1	rs1443707	intron_variant	G	A	calpain 14	0.0286
chr2	31400722	ENSG00000214711	*CAPN14*	2p23.1	rs1443706	intron_variant	G	A	calpain 14	0.0286
chr2	31400867	ENSG00000214711	*CAPN14*	2p23.1	rs1373216	intron_variant	T	C	calpain 14	0.0286
chr2	31401499	ENSG00000214711	*CAPN14*	2p23.1	rs28684727	intron_variant	G	A	calpain 14	0.0286
chr2	31403947	ENSG00000214711	*CAPN14*	2p23.1	rs2028678	intron_variant	G	A	calpain 14	0.0286
chr2	174946760	ENSG00000138430	*OLA1*	2q31.1	rs11558990	non-synonymous SNV	T	C	Obg-like ATPase 1	0.0286
chr2	174988189	ENSG00000138430	*OLA1*	2q31.1	rs10930639	intron_variant	C	T	Obg-like ATPase 1	0.0286
chr2	175199895	ENSG00000231453	*AC018470.4*	2q31.1	rs3856434	downstream_gene_variant	G	A	Sp9 transcription factor	0.0286
chr3	42772038	ENSG00000244607	*CCDC13*	3p22.1	rs12495805	non-synonymous SNV	A	T	coiled-coil domain containing 13	0.0286
chr3	124646594	ENSG00000173702	*MUC13*	3q21.2	rs4679394	non-synonymous SNV	A	G	mucin 13, cell-surface-associated	0.0286
chr3	190967779	ENSG00000188729	*OSTN*	3q28	rs2034771	intron_variant	A	G	osteocrin	0.0286
chr4	91645179	ENSG00000184305	*CCSER1*	4q22.1	rs62314447	intron_variant	A	T	multimerin 1	0.0286
chr6	47253631	ENSG00000146072	*TNFRSF21*	6p12.3	rs11758366	intron_variant	A	G	tumor necrosis factor receptor superfamily member 21	0.0286
chr7	3861353	ENSG00000146555	*SDK1*	7p22.2	rs6943646	intron_variant	C	G	sidekick cell adhesion molecule 1	0.0286
chr7	72396170	ENSG00000196313	*POM121*	7q11.23	rs782134793	intron_variant	GCGCCGCG CTCCCCAC	G	POM121 transmembrane nucleoporin	0.0286
chr7	140036999	ENSG00000157800	*SLC37A3*	7q34	rs4332050	intron_variant	G	A	solute carrier family 37 member 3	0.0286
chr7	140044979	ENSG00000157800	*SLC37A3*	7q34	rs6974016	upstream_gene_variant	C	T	solute carrier family 37 member 3	0.0286
chr9	100889340	ENSG00000106789	*CORO2A*	9q22.33	rs942165	intron_variant	G	T	coronin 2A	0.0286
chr10	51549314	ENSG00000138294	*MSMB*	10q11.23	rs12770171	upstream_gene_variant	C	T	translocase of inner mitochondrial membrane 23 homolog B	0.0286
chr10	129179426	ENSG00000150760	*DOCK1*	10q26.2	rs7099958	intron_variant	T	C	dedicator of cytokinesis 1	0.0286
chr11	3078536	ENSG00000110619	*CARS*	11p15.4	rs4758463	intron_variant	C	G	cysteinyl-tRNA synthetase	0.0286
chr12	122079189	ENSG00000182500	*ORAI1*	12q24.31	rs3741595	synonymous_variant	C	T	ORAI calcium release-activated calcium modulator 1	0.0286
chr12	131623850	ENSG00000111452	*ADGRD1*	12q24.33	rs35160436	non-synonymous SNV	A	AC	adhesion G protein-coupled receptor D1	0.0286
chr13	113793849	ENSG00000126218	*F10*	13q34	rs3211770	upstream_gene_variant	G	A	coagulation factor X	0.0286
chr14	35228090	ENSG00000198604	*BAZ1A*	14q13.1	rs61981202	intron_variant	G	A	bromodomain adjacent to zinc finger domain 1A	0.0286
chr14	35237874	ENSG00000198604	*BAZ1A*	14q13.1	rs61981228	downstream_gene_variant	C	A	bromodomain adjacent to zinc finger domain 1A	0.0286
chr14	35483882	ENSG00000100883	*SRP54*	14q13.2	rs13379372	sequence_feature	A	C	signal recognition particle 54kDa	0.0286
chr14	35492299	ENSG00000100883	*SRP54*	14q13.2	rs4982254	upstream_gene_variant	AG	A	signal recognition particle 54kDa	0.0286
chr14	35492301	ENSG00000100883	*SRP54*	14q13.2	rs80306194	upstream_gene_variant	CTTGTTATT AGTTAACAG	C	signal recognition particle 54kDa	0.0286
chr14	35497285	ENSG00000100883	*SRP54*	14q13.2	rs78609489	intron_variant	T	C	signal recognition particle 54kDa	0.0286
chr16	2906934	ENSG00000263325	*LA16c-325D7.1*	16p13.3	rs732532	upstream_gene_variant	G	A	protease, serine 22	0.0286
chr16	3021417	ENSG00000127564	*PKMYT1*	16p13.3	rs79505645	upstream_gene_variant	G	T	progestin and adipoQ receptor family member IV	0.0286
chr16	15126890	ENSG00000179889	*PDXDC1*	16p13.11	rs12926897	upstream_gene_variant	C	T	pyridoxal-dependent decarboxylase domain containing 1	0.0286
chr16	15850204	ENSG00000133392	*MYH11*	16p13.11	rs2272554	synonymous_variant	A	G	myosin, heavy chain 11, smooth muscle	0.0286
chr16	15853596	ENSG00000133392	*MYH11*	16p13.11	rs2280764	intron_variant	C	G	myosin, heavy chain 11, smooth muscle	0.0286
chr16	16138322	ENSG00000103222	*ABCC1*	16p13.11	rs246221	synonymous_variant	T	C	ATP binding cassette subfamily C member 1	0.0286
chr16	16139714	ENSG00000103222	*ABCC1*	16p13.11	rs35587	synonymous_variant	T	C	ATP binding cassette subfamily C member 1	0.0286
chr16	16139878	ENSG00000103222	*ABCC1*	16p13.11	rs35588	splice_region_variant and intron_variant	A	G	ATP binding cassette subfamily C member 1	0.0286
chr17	57951973	ENSG00000108423	*TUBD1*	17q23.1	rs2250526	synonymous_variant	G	A	tubulin delta 1	0.0286
chr17	57992145	ENSG00000241913	*RP5-1073F15.1*	17q23.1	rs3066247	downstream_gene_variant	TATC	T	ribosomal protein S6 kinase B1	0.0286
chr17	58037374	ENSG00000189050	*RNFT1*	17q23.1	rs12600680	upstream_gene_variant	T	C	ring finger protein, transmembrane 1	0.0286
chr17	58042126	ENSG00000189050	*RNFT1*	17q23.1	rs76419616	upstream_gene_variant	T	C	TBC1D3P1-DHX40P1 readthrough, transcribed pseudogene	0.0286
chr19	33882222	ENSG00000124299	*PEPD*	19q13.11	rs17569	synonymous_variant	G	A	peptidase D	0.0286
chr22	23487533	ENSG00000100228	*RAB36*	22q11.22	rs1476441	5_prime_UTR_variant	C	T	RAB36, member RAS oncogene family	0.0286

**Table 4:** Gene Set Enrichment Analysis (GSEA) for *ALPK2*.

GeneSets	NES	NOM p-val	FDR q-val
Reactome WNT Ligand Biogenesis and Trafficking	1.41	0.110	0.398
PID WNT Signaling Pathway	1.40	0.104	0.221
WNT Up.V1 Up	1.15	0.249	0.356
WNT Up.V1 DN	0.90	0.566	0.582
Reactome Beta Catenin Independent WNT Signaling	−3.18	0.000	0.000
Reactome Signaling By WNT	−3.07	0.000	0.000
WP WNT Signaling Pathway	−2.07	0.004	0.014
Hallmark WNT Beta Catenin Signaling	−2.06	0.004	0.012
PID WNT Canonical Pathway	−1.97	0.010	0.014
Biocarta WNT Pathway	−1.74	0.023	0.043
PID WNT Noncanonical Pathway	−1.70	0.025	0.045
KEGG WNT Signaling Pathway	−1.54	0.050	0.081
WP WNT Signaling	−1.26	0.185	0.235
WNT Signaling	−1.21	0.241	0.257
Reactome Signaling by WNT In Cancer	−1.20	0.242	0.241
GOCC Catenin Complex	1.82	0.012	0.066
GOMF WNT Protein Binding	0.90	0.586	0.736
GOBP Cell Cell Signaling By WNT	−2.61	0.000	0.000
GOBP Regulation of WNT Signaling Pathway	−2.24	0.000	0.003
HP Downturned Corners of Mouth	−2.17	0.000	0.005
GOBP Positive Regulation of WNT Signaling Pathway	−2.06	0.000	0.009
GOBP Canonical WNT Signaling Pathway	−2.05	0.000	0.010
GOBP Negative Regulation of Canonical WNT Signaling Pathway	−1.94	0.008	0.017
GOBP Negative Regulation of WNT Signaling Pathway	−1.80	0.011	0.032
GOBP Positive Regulation of Canonical WNT Signaling Pathway	−1.77	0.021	0.036
GOMF Beta Catenin Binding	−1.55	0.057	0.090
GOBP Non-canonical WNT Signaling Pathway	−1.51	0.063	0.105
GOBP Regulation of Non-canonical WNT Signaling Pathway	−1.25	0.195	0.253
GOMF WNT Receptor Activity	−1.17	0.237	0.315
GOBP Regulation of WNT Signaling Pathway Planner Cell Polarity Pathway	0.96	0.487	0.544

NES = Normalized enriched score; NOM p-val = Sta:s:cally significant pathways *p* < 0.05; FDR qval = FDR adjusted *p*-value < 0.05.

## Data Availability

Data supporting the findings of this study are available from the corresponding author upon reasonable request. The data are not publicly available due to privacy.

## Supplementary Materials

The following supporting information can be downloaded at: https://www.mdpi.com/article/10.3390/genes15030357/s1.

## Abbreviations

NAFLD: non-alcoholic fatty liver disease; NASH, non-alcoholic steatohepatitis; MASLD, metabolic dysfunction-associated fatty liver disease; MASH, metabolic dysfunction-associated fatty liver disease; WES, whole-exome sequencing; SNP, single nucleotide polymorphism; nsSNV, non-synonymous single nucleotide variant; InDels, insertions and deletions; HCC, hepatocellular carcinoma; ALPK2, α kinase 2; GSEA, gene set enrichment analysis; FACETS, fraction and allele-specific copy number estimates from tumor sequencing; PCR, polymerase chain rection.
