# Supplementary material for: Whole-Exome Sequencing (WES) Reveals Novel Sex-Specific Gene Variants in Non-Alcoholic Steatohepatitis (MASH)
Source: Genes (Basel). 2024 Mar 13;15(3):357. doi: 10.3390/genes15030357 (PMC10969913; doi:10.3390/genes15030357)
Supplement: Supplementary file 1 [file genes-15-00357-s001.zip › genes-2893311-supplementary.pdf]

Table S1. Clinicopathological characteristics of normal and NASH cohorts. Total (n=16)

Parameter	Male		Female	
	Normal	NASH	Normal	NASH
Number of cases	4	4	4	4
Mean age, years, SD	54 ±12.5	56±11.3	58.4±13.3	59.5±14.5
Race (%)	White (100)	White (100)	White (100)	White (100)
Diabetes mellitus, %	75	75	75	100
Obesity, %	50	75	75	100
BMI, kg/m^2, SD	29.6±5.6	31±6.4	30.3±6.1	35.3±6.3
AST, U/L	12±2.6	67±11.8	26±4.7	88±14.1
ALT, U/L	44±5.1	92±10.2	52±6.1	110±10.6
Steatosis, %	<0.1	100	<0.1	100
Steatohepatitis, %	<0.1	75	<0.1	100
Ballooning, %	<0.1	50	<0.1	100
Portal Inflammation, %	<0.1	75	<0.1	100
Fibrosis, %	<0.1	50	<0.1	75

Figure S1

A)

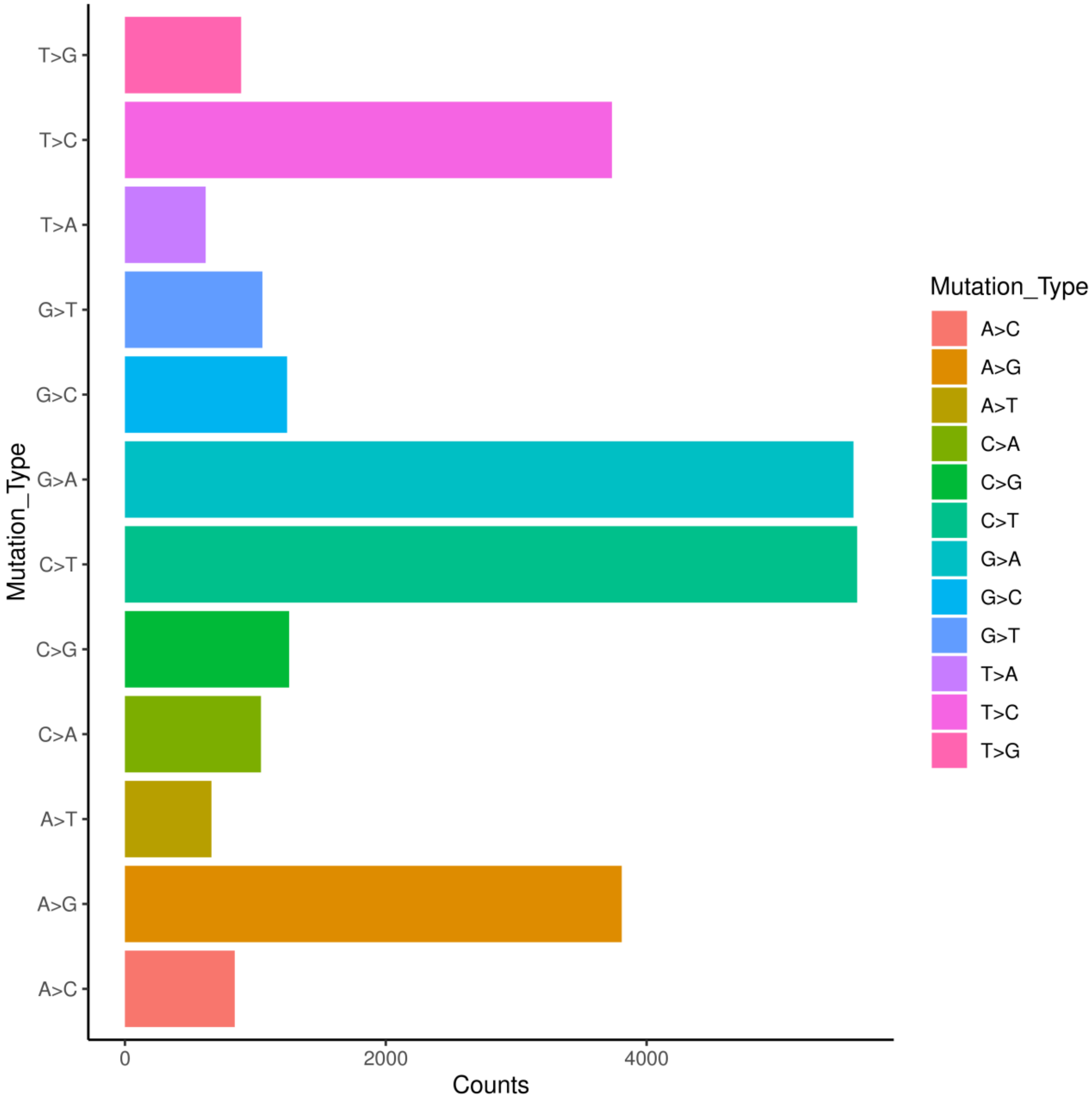
Mutation_Type
T>G
T>C
T>A
G>T
G>C
G>A
C>T
C>G
C>A
A>T
A>G
A>C
0
2000
4000
Counts
Mutation_Type
A>C
A>G
A>T
C>A
C>G
C>T
G>A
G>C
G>T
T>A
T>C
T>G

Figure S1

B)

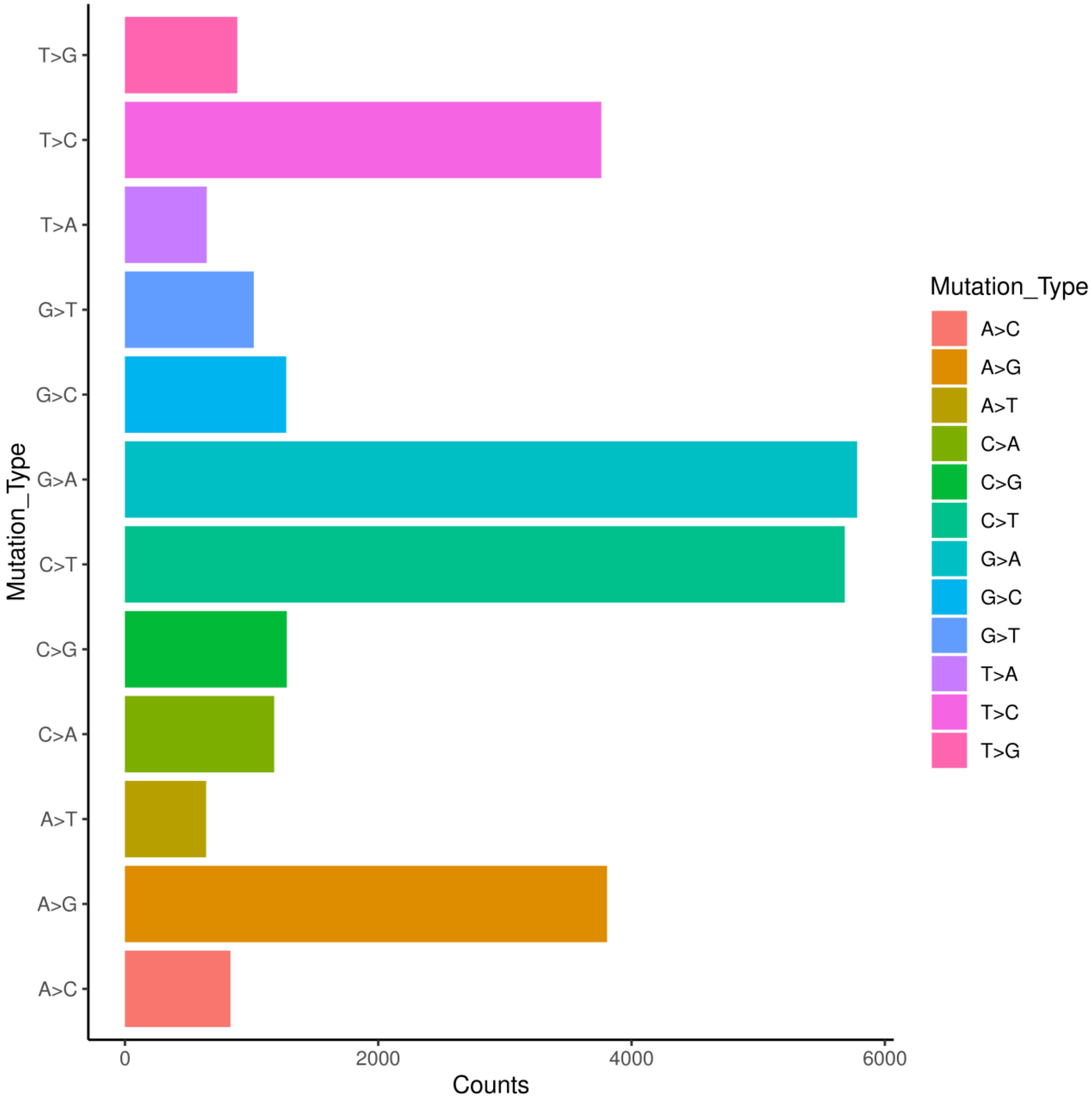

Figure S1. Representative of mutation spectrum analysis for NASH-associated female (A) and male (B) liver tissue samples.

Figure S2

A)

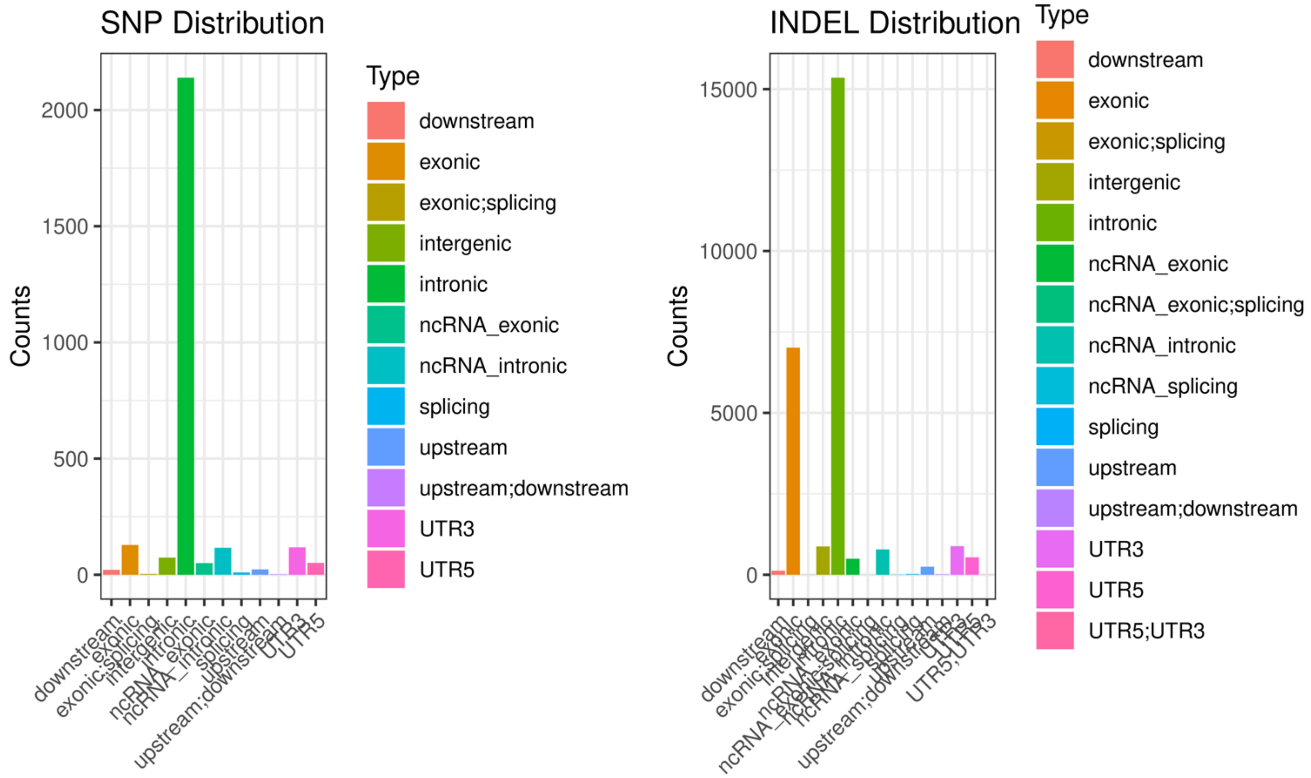
SNP Distribution
Counts
2000
1500
1000
500
0
Type
downstream
exonic
exonic;splicing
intergenic
intronic
ncRNA_exonic
ncRNA_intronic
splicing
upstream
upstream;downstream
UTR3
UTR5
INDEL Distribution
Counts
15000
10000
5000
0
Type
downstream
exonic
exonic;splicing
intergenic
intronic
ncRNA_exonic
ncRNA_exonic;splicing
ncRNA_intronic
ncRNA_splicing
splicing
upstream
upstream;downstream
UTR3
UTR5
UTR5;UTR3

Figure S2
B)

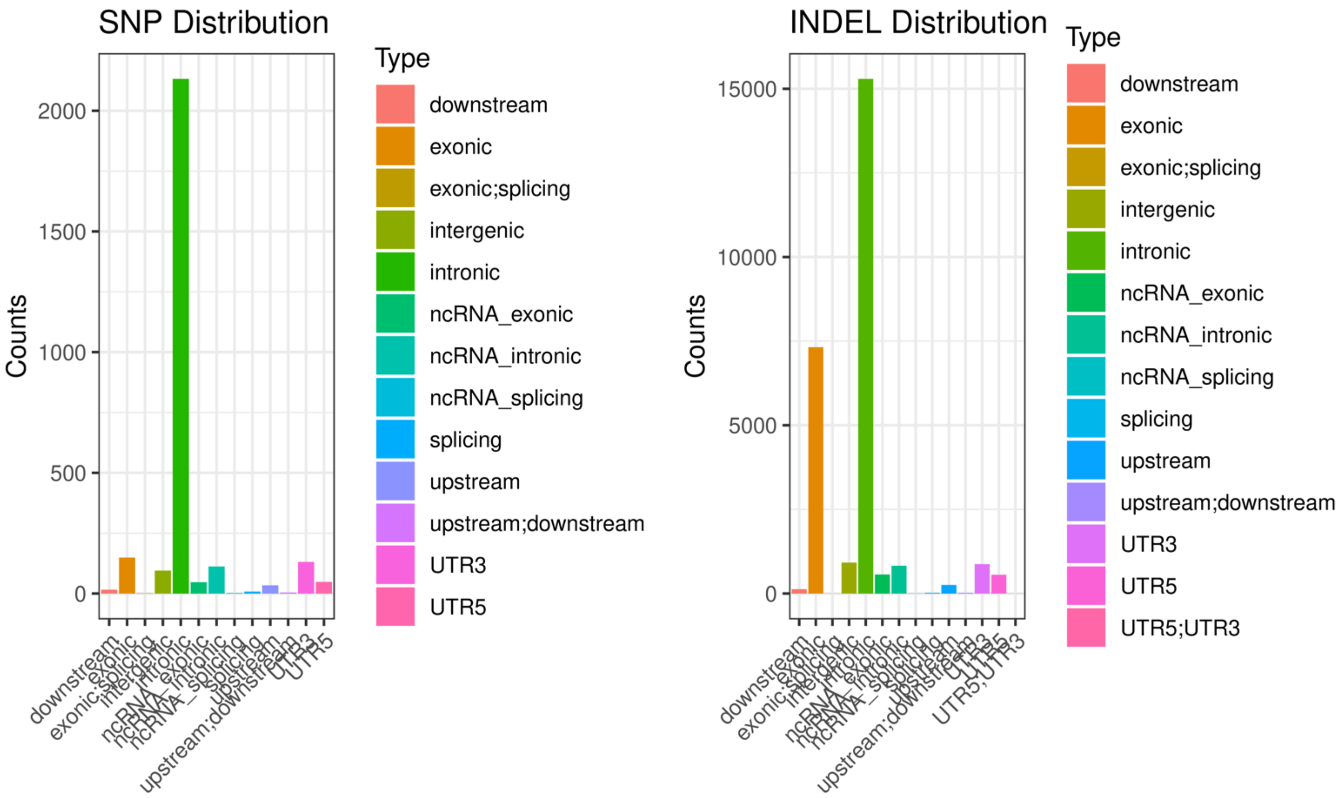

Figure S2. Representative of SNP and InDels spectrum analysis for NASH-associated female (A) and male (B) liver tissue samples.
